# Cardiomyocyte-targeted and 17β-estradiol-loaded acoustic nanoprobes as a theranostic platform for cardiac hypertrophy

**DOI:** 10.1186/s12951-018-0360-3

**Published:** 2018-03-30

**Authors:** Xueli Zhao, Wen Luo, Jing Hu, Lei Zuo, Jing Wang, Rui Hu, Bo Wang, Lei Xu, Jing Li, Meng Wu, Pan Li, Liwen Liu

**Affiliations:** 1 Department of Ultrasound, Xijing Hypertrophic Cardiomyopathy Center, Xijing Hospital, Fourth Military Medical University, Xi’an, 710032 China; 2 Department of Stomatology, PLA Army General Hospital, Beijing, 100700 China; 3 Department of Ultrasound, Zhongnan Hospital of Wuhan University, Wuhan, 430071 China; 4 Second Affiliated Hospital of Chongqing Medical University & Chongqing Key Laboratory of Ultrasound Molecular Imaging, Chongqing, 400010 China

**Keywords:** Theranostic nanoprobes, Low-intensity focused ultrasound, Cardiac targeting, Cardiac hypertrophy, 17β-estradiol

## Abstract

**Background:**

Theranostic perfluorocarbon nanoprobes have recently attracted attention due to their fascinating versatility in integrating diagnostics and therapeutics into a single system. Furthermore, although 17β-estradiol (E2) is a potential anti-hypertrophic drug, it has severe non-specific adverse effects in various organs. Therefore, we have developed cardiomyocyte-targeted theranostic nanoprobes to achieve concurrent targeted imaging and treatment of cardiac hypertrophy.

**Results:**

We had successfully synthesized E2-loaded, primary cardiomyocyte (PCM) specific peptide-conjugated nanoprobes with perfluorocarbon (PFP) as a core (PCM-E2/PFPs) and demonstrated their stability and homogeneity. In vitro and in vivo studies confirmed that when exposed to low-intensity focused ultrasound (LIFU), these versatile PCM-E2/PFPs can be used as an amplifiable imaging contrast agent. Furthermore, the significantly accelerated release of E2 enhanced the therapeutic efficacy of the drug and prevented systemic side effects. PCM-E2/PFPs + LIFU treatment also significantly increased cardiac targeting and circulation time. Further therapeutic evaluations showed that PCM-E2/PFPs + LIFU suppressed cardiac hypertrophy to a greater extent compared to other treatments, revealing high efficiency in cardiac-targeted delivery and effective cardioprotection.

**Conclusion:**

Our novel theranostic nanoplatform could serve as a potential theranostic vector for cardiac diseases.

**Electronic supplementary material:**

The online version of this article (10.1186/s12951-018-0360-3) contains supplementary material, which is available to authorized users.

## Background

Cardiac hypertrophy is a progressively pathological and compensatory reaction to chronic pressure overload that is characterized by left ventricular (LV) dysfunction, cardiomyocyte hypertrophy and interstitial fibrosis, which may eventually lead to heart failure and sudden death [1, 2]. Moreover, patients with cardiac hypertrophy do not show typical clinical symptoms during the early stages, making early clinical diagnosis and effective treatment difficult. As cardiac hypertrophy progresses, improved diagnostic and therapeutic strategies are imperative for early detection, treatment, and follow-up, thus preventing the occurrence of irreversible damage.

The emergence of theranostics, a fascinating tool that integrates diagnostics and therapeutics into a single procedure, provides good strategies for monitoring diseases, optimizing drug efficacy, and reducing the side effects of various drugs [3, 4], which result in significant cost savings for the patients [5].

During the last decade, microbubble contrast agents have been the pioneers of cocktail therapeutic agents for both diagnosis and treatment [6–8]. However, their micron size and limited circulatory life span in vivo (a few minutes) have resulted in extravascular imaging limitations and insufficient bubble accumulation in targeted sites, which necessitated high dosages or multiple injections during theranostics [9–11]. Recently emerging acoustic phase-change nanodroplets have sparked interest for their theranostic capabilities in ultrasound (US) imaging and therapeutic applications. Compared to conventional microbubbles, they had larger nanometer size, better stability, and longer circulation time [12], making them more appropriate theranostic agents. Moreover, they were able to immediately convert into microbubbles from an initially liquid state once exposed to sufficient US pressures at desired positions [13–16]. The generated bubbles can produce strong echogenicity in local tissues for US imaging, while this “small-to-big” process pave the way for drug release from the nanodroplets [14]. However, this theranostic probe still faces challenges in achieving increased theranostic specificity during targeted imaging and therapy.

Attaching specific targeting moieties (e.g., antibodies, aptamers, and peptides) to the nanoprobe surface provides the theranostic nanodroplets with the robust ability for targeted US imaging and drug delivery [17–19]. PCM, a phage display-isolated 20-mer peptide (WLSEAGPVVTVRALRGTGSW) with excellent cardiomyocyte specificity [20], can be conjugated to the nanodroplet surface to further increase its cardiac-targeting ability [21, 22], making it an ideal targeting moiety for active cardiac nanoprobe delivery.

Estrogen, especially 17β-estradiol (E2), has been suggested to exert significant anti-hypertrophic action. Clinical studies have shown that gender influences patterns of LV hypertrophy [23], while postmenopausal women have higher incidences of cardiac hypertrophy relative to age-matched men [24]. Various animal studies have also supported the protective actions of estrogen against cardiac hypertrophy. For example, Li et al. have shown that estrogen can prevent overload-induced hypertrophy by inhibiting mast cell chymase release [25]. Furthermore, estrogen can prevent cardiac fibrosis and heart failure [26, 27], which are major factors in the development of cardiac hypertrophy. All available evidence has shown that estrogen plays a potentially important role in the treatment and prevention of cardiac hypertrophy. Unfortunately, long-term, high-dose usage of estrogen has been known to cause severe adverse reactions elsewhere in the body, such as feminization in males, breast and ovarian cancers, uterine bleeding, and hyperplasia [28]. In addition, because of the hepatic first-pass metabolism resulting from its common oral administration route, high doses are required to achieve a therapeutic effect. This, accompanied by nonspecific accumulation of most drugs in other tissues, results in increased adverse effects and weakened functional efficacy [29]. These significant drawbacks hinder the clinical use of E2 in the treatment of cardiac hypertrophy. Therefore, the development of effective tools for improving therapeutic effects while reducing side effects is essential for the successful application of this drug.

In this study, we developed a novel theranostic probe that is capable of cardiac-targeted imaging and treatment. We synthesized targeting theranostic nanoprobes through PCM conjugation, E2-loaded, and PFP encapsulation (PCM-E2/PFPs), and demonstrated their capability for targeted US imaging, intrinsic preferential cardiac accumulation, and cardiac hypertrophy treatment while reducing undesired side effects with the assistance of LIFU. Therefore, the well-defined dual-responsive PCM-E2/PFPs probe has a strong potential for clinical application in humans.

## Methods

PCM (WLSEAGPVVTVRALRGTGSW) and FITC-PCM were purchased from GL Biochem Ltd. (Shanghai). PLGA-COOH (50:50, MW ¼ 20,000) was obtained from Jinan Daigang Biomaterial Co., Ltd. (China). Perfluoropentane (PFP), MES hydrate, 1-ethyl-3-(3-dimethylaminopropyl)-carbodiimide hydrochloride (EDC), and *N*-hydroxysuccinimide (NHS) fluorescent dyes, including 4′,6-diamidino-2-phenylindole (DAPI) and 1,1′-dioctadecyl-3,3,3′,3′-tetramethylindocarbocyanine perchlorate (DiI), were obtained from Sigma-Aldrich Chemical Co. (St. Louis, MO, USA). 17β-estradiol (E2) was purchased from Solarbio (China). Deionized water was used in all experiments.

### Preparation of PCM-conjugated and E2-loaded PFP nanoprobes (PCM-E2/PFPs)

Targeting PCM-E2/PFPs nanodroplets were fabricated using a method previously described [30]. Briefly, 100 mg PLGA-COOH and 10 mg E2 were completely dissolved in 2 mL dichloromethane (CH_2_Cl_2_) (to prepare fluorescent nanoprobes, a few drops of DiI fluorescent dye were added to this solution). Thereafter, 200 μL PFP was slowly poured into the polymer solution, which was then emulsified using an ultrasonic probe (Sonics & Materials Inc., USA) at 250 W for 4 min (5 s on/5 s off vibration cycle to prevent phase transition). The above-described emulsified solution was then poured into 10 mL PVA (4% w/v) solution and homogenized (FJ300-SH, Shanghai, China) for 5 min in preparation for another emulsion. The final emulsion with 15 mL 2% (w/v) isopropyl alcohol solution, which was added to remove the foam, was stirred with a magnetic stirrer (HJ-1, Ronghua, China) for 6 h to remove CH_2_Cl_2_. Subsequently, the solution was cryogenically centrifuged at 8000 rpm for 5 min. Finally, the supernatant was discarded and the precipitate was washed thrice with deionized water, and non-targeting E2/PFPs were prepared.

Conjugation of PCM peptides to the E2/PFPs surface was performed based on a method used for carbodiimides. The prepared E2/PFPs were dispersed in 10 mL MES buffer (0.1 mol/L, pH = 5.5) together with 12 mg EDC and 8 mg NHS for oscillation and incubated for 30 min. Residual EDC and NHS were extracted using MES buffer (0.1 mol/L, pH = 5.5) after being centrifuged three times for 5 min at 8000 rpm. The precipitate was then dissolved using MES buffer (0.1 mol/L, pH = 8.0), and 1 mL PCM peptides solution (1 mg/mL) were dropped into above solution followed by incubation for 2 h at 4 °C with continuous shaking. Subsequently, after three consecutive centrifugations and PCM-E2/PFPs harvesting, all aforementioned steps were manipulated at 4 °C. Pure nanoprobes (PCM-E2/H_2_Os) were prepared similarly using 200 μL deionized water instead of 200 μL PFP.

### Characterization of PCM-E2/PFPs

To better understand the characterization of the prepared nanoprobes, a transmission electron microscope (H7600; Hitachi, Japan) was used to observe the nanodroplet morphology, with the nanodroplets being carefully dropped onto a copper grid and negatively stained. The particle size and zeta potential of the nanodroplets were analyzed at 25 °C through dynamic light scattering (DLS) using a laser particle size analyzer (Zeta SIZER 3000HS; Malvern, USA). Additionally, PCM-E2/PFPs were stored at 4 °C, while the mean particle size was measured at different time points after preparation (12 h and 1, 2, 3, 4, and 5 days) to evaluate the stability of the nanodroplets.

PCM conjugation efficiency was determined by measuring the fluorescence of the FITC-labeled PCM and DiI-labeled E2/PFPs using confocal laser scanning microscopy (CLSM) (A1R; Nikon, Japan) and flow cytometry (BD Influx, BD, USA). The temperature-responsive phase transition process of PCM-E2/PFPs was observed at 200× magnification using an inverted fluorescence microscope (CKX41; Olympus, Japan) while increasing the temperature from 25 to 60 °C using a heating panel.

### E2 release behavior of LIFU-triggered and temperature-dependent PCM-E2/PFPs in vitro

First, the temperature variation of the nanoprobes upon LIFU irradiation was measured. 1 mL PCM-E2/PFPs (10 mg/mL) solution was placed into a dialysis membrane (Mw = 3500 Da), then irradiated with LIFU (2.4 W/cm^2^) for 0, 3, 5, 10, 15, 20 min and then the temperature of the nanodroplets at different times were measured with a thermometer.

Then to evaluate PCM-E2/PFPs drug release with LIFU irradiation or heated separately, 1 mL PCM-E2/PFPs (10 mg/mL) solution was placed into a dialysis membrane, after LIFU irradiation (LM.SC051 ACA; Institute of Ultrasound Imaging of Chongqing Medical Sciences, China) for 10 min at 2.4 W/cm^2^ or heated for 10 min at 45 °C, then submerged in 30 mL PBS/Tween 80 solution at 37 °C (simulating the body temperature), the buffer (1 mL) was sampled periodically at fixed intervals and was replenished with an equal volume thereof. The amount of E2 release from the PCM-E2/PFPs was analyzed using high-performance liquid chromatography (HPLC) (Agilent 1100; Agilent, USA) equipped with a C18 column at 30 °C. The accumulative ratios of released E2 were calculated at different intervals. The aforementioned steps were performed for controls except for LIFU irradiation or heated.

### Evaluation of LIFU-triggered US imaging ability in vitro and in vivo

All US images in vitro and in vivo were obtained using MyLab 90 (Esaote, Italy) with a linear probe (5–12 MHz). To evaluate US imaging capacity in vitro, agar gel phantom was made using 3% agar–agar (w/v) dissolved in deionized water using a 2 mL eppendorf tube model to mimic conditions similar to those in vivo. After treatment at different LIFU frequencies (1.2, 1.8, 2.4, and 3.2 W/cm^2^) with sonovue as the control, US images were captured under standard B-mode and contrast mode using the same instrument parameters. Mean echo intensities of the captured images were then analyzed quantitatively using DFY (Invented by the Institution of Ultrasound Imaging of Chongqing Medical University, Chongqing, China).

For in vivo US imaging, normal Sprague–Dawley rats were anesthetized and intravenously injected with PCM-E2/PFPs or E2/PFPs via the tail vein, while controls were injected with sonovue. LIFU irradiation (3.2 W/cm^2^, 10 min) was performed after injection. Subsequently, US images of the heart were observed at different intervals after LIFU sonication. The obtained US intensity was analyzed using the same method described previously.

### Assessment of PCM-E2/PFP biodistribution in vivo

Rats were divided into three groups (n = 8): targeting PCM-E2/PFPs group, targeting PCM-E2/PFPs + LIFU group, and non-targeting E2/PFPs + LIFU group, while the distribution of PCM-E2/PFPs vesicles after treatment was tracked using the fluorescence signal of DiI on the vesicle shell. All animals were anesthetized with an intraperitoneal injection of 1% pentobarbital (40 mg/kg) followed by DiI-labeled nanodroplet injection (0.4 mg/kg) via the tail vein. LIFU (3.2 W/cm^2^, 10 min) was applied 3 min post-injection. Hearts and other major organs of PCM-E2/PFPs + LIFU treated rats were extracted 10 h after the injection to evaluate the targeting ability of PCM-E2/PFPs. Moreover, hearts from the PCM-E2/PFPs and E2/PFPs + LIFU groups were obtained to evaluate LIFU-triggered cardiac accumulation behavior of the nanoprobes. All tissue samples were frozen, while 4–5 μm cryosections were continuously cut under − 20 °C conditions. After fixation for 15 min using 4% paraformaldehyde, DAPI dying was done for 10 min in the dark. Sealed slides of tissue sections were used for CLSM analysis. Meanwhile, serum samples of PCM-E2/PFPs + LIFU were collected after periodic intervals, and DiI concentration from serum samples was assayed using fluorometry at Ex 549 nm/Em 565 nm.

### Treatment with in vivo therapeutics

#### Ovariectomy (OVX) and transverse aortic constriction (TAC) (animal preparation)

Female Sprague–Dawley rats (200–250 g) were housed under optimum conditions and anesthetized in preparation for bilateral ovariectomy. After a 1 week recovery period, a cardiac hypertrophy model was generated using TAC on OVX rats [31]. Sham groups underwent a similar surgical procedure without aortic ligation.

#### Animal groups and treatments

To verify the treatment efficacy of PCM-E2/PFPs + LIFU, all OVX rats were randomized into the following six groups (n = 8):

1. Sham group: OVX rats without aortic ligation that were treated with normal saline.
2. TAC group: OVX rats that underwent TAC surgery and were treated with normal saline.
3. E2/PFPs group: OVX rats that underwent TAC surgery and were treated with non-targeting E2/PFP solution without LIFU irradiation.
4. PCM-E2/PFPs group: OVX rats that underwent TAC surgery and were treated with targeting PCM-E2/PFP solution without LIFU irradiation.
5. E2/PFPs + LIFU group: OVX rats that underwent TAC surgery and were treated with non-targeting E2/PFP solution with LIFU irradiation.
6. PCM-E2/PFPs + LIFU group: OVX rats that underwent TAC surgery and were treated with targeting PCM-E2/PFPs solution with LIFU irradiation.

Treatment began after a recovery period of 1 week and was conducted once every 3 days for 6 consecutive weeks. LIFU irradiation (3.2 W/cm^2^, 10 min) was performed on the cardiac sites 10 min, 30 min, and 1 h after nanodroplet injection to allow more nanodroplets to refill the heart. Groups (3) to (6) received drugs containing an E2 nanodroplet solution (0.4 mg/kg) prior to LIFU treatment. At the endpoint of the experiments, the rats were sacrificed and then the wet weights of the hearts (HW) and left ventricle (LW) were determined and normalized by the tibia length (TL). LV tissue samples were fixed in 10% formalin for histological analysis, while the remaining portions were snap frozen in liquid nitrogen for subsequent Reverse transcription PCR (RT-PCR) analysis. To evaluate potential in vivo toxicity, the major organs, including the brain, lungs, liver, spleen, and kidneys, from the TAC and PCM-E2/PFPs + LIFU groups were harvested for hematoxylin and eosin (HE) staining. Serum samples from the same groups were also collected for biochemical examination of liver (ALT, alanine transaminase; ALP, alkaline phosphates; AST, aspartate aminotransferase) and renal (Cr, creatinine; BUN, blood urea nitrogen; UA, uric acid) function assays using and automatic biochemical analyzer (Chemray 240; Rayto, China).

### Transthoracic echocardiography

Anesthetized experimental rats were analyzed using serial B-Mode and M-Mode echocardiography to assess LV function. Interventricular septum end-diastolic thickness (IVSD), posterior end-diastolic wall thickness (LVPWd), and end-diastolic diameter (LVDD) were measured from LV M-mode images. Ejection fraction (EF) was calculated using VisualSonics Measurement Software formulae.

### HE and Masson’s staining

Paraffin-embedded, 4–5 μm thick transversal sections from formalin-fixed LV tissues were prepared and stained with HE and Masson’s Trichrome for histopathology and collagen deposition, respectively, according to manufacturer’s protocol. Digital pictures were taken using light microscopy with identical exposure settings for all sections. The average cross sectional area (CSA) was measured from an area of selected myocytes using ImageJ (NIH), whereas cardiac collagen volume fraction (CVF) was quantified using Image-Pro plus 6.0 imaging software.

### Reverse transcription PCR (RT-PCR)

Total RNA from the frozen LV tissues was extracted using TRIzol (Invitrogen), while RNA purity and concentration were assessed using a spectrophotometer (A260/A280) (Beckman). After 2 μg of total RNA were reverse transcribed into cDNA using the PrimeScript™ RT Reagent Kit (TAKARA), RT-PCR of target genes was performed using specific SYBR^®^ Premix Ex Taq™ II (Tli RNaseH Plus) with a 7500 Fast Real-Time PCR System (Applied Biosystems, Foster City, CA, USA). All reactions were done in a final volume of 20 μL following the manufacturer’s instructions. Levels of target gene expression were determined using the comparative Ct method, the relative amounts of which were normalized to GAPDH mRNA. Primer sequences are listed in Table 1.Controls were prepared using the same reaction, except for the absence of reverse transcription and the use of H_2_O instead of cDNA for the RT-qPCR test.

**Table 1:** Sequences of primers for RT-PCR

Gene	Forward primer (5′–3′)	Reverse primer (5′–3′)
GAPDH	CGGGAAATCGTGCGTGAC	TCGCTCCAACCGACTGCT
β-MHC	ACCAGTCCATCCTCATCACC	TGGCAGCAATAACAGCAAAA
Collagen 1	ACTCATGGCCAAGAAGACATC	TTTGCATAGCACGCCATCG
Collagen 3	TTTGGCACAGCAGTCCAATG	TCCCGAGTCGCAGACACATAT

### Statistical evaluation

All statistical evaluations were carried out using one-way analysis of variance, with data being expressed as mean ± SEM. Experiments were repeated at least three times before analysis. A p-value smaller than 0.05 was considered indicative of statistical significance.

## Results and discussion

**Fig. 1:**
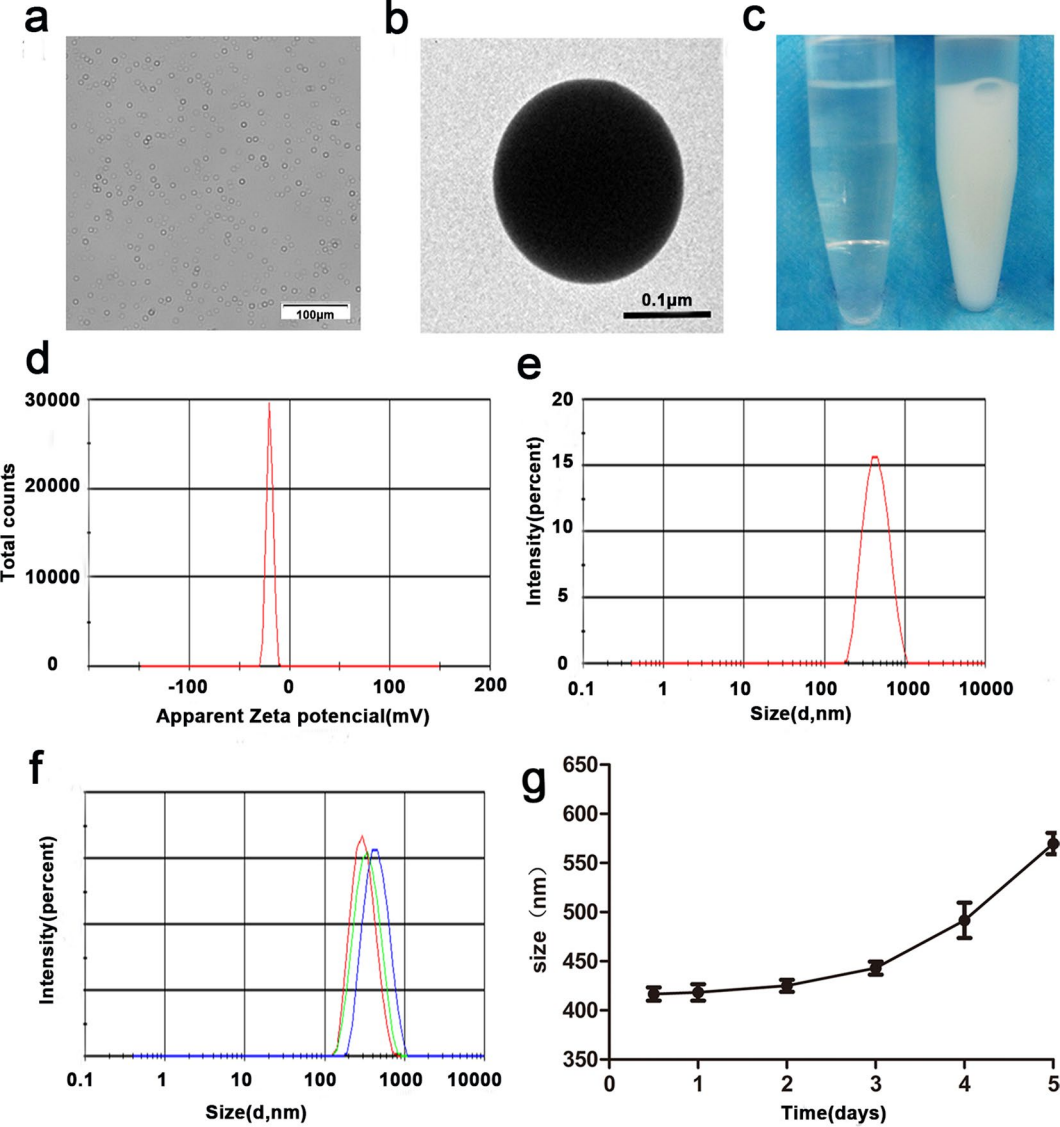
Characterizations of PCM-E2/PFPs. **a** Optical microscopy image of PCM-E2/PFPs. b TEM image of PCM-E2/PFPs. **c** Macroscopic views of free PFP and PCM-E2/PFPs at the same PFP concentration in PBS. **d**, **e** Size distribution and Zeta potential of PCM-E2/PFPs. **f** Size distributions of E2/H_2_Os, PCM-E2/H_2_Os and PCM-E2/H_2_Os. **g** Size changes of PCM-E2/PFPs at 4 °C after long-term storage (n = 3)

We produced PCM-E2/PFPs nanoprobes through PCM conjugation, E2-loaded, and PFP encapsulation using a typical two-step emulsion process. A low-temperature process had to be adopted due to the relatively low boiling point of PFP (29 °C). As shown in Fig. 1, PCM-E2/PFPs nanodroplets had a milky white appearance (Fig. 1c) and presented an almost perfectly spherical morphology (Fig. 1a, b). It had an average diameter of 418 ± 11 nm with homogeneous distribution, as well as an average surface zeta potential of − 20 ± 1 mV (Fig. 1d, e). In addition, size distributions of different nanodroplets were compared to evaluate the effects of conjugation and encapsulation. We found no significant differences (p > 0.05) in size distributions between non-targeting E2 nanodroplets encapsulated with saline (E2/H_2_Os) and targeting PCM-conjugated E2 nanodroplets encapsulated with saline (PCM-E2/H_2_Os). After the addition of PFP to the targeting E2 nanodroplets (PCM-E2/PFPs), an apparent increase in average size was observed, although homogeneity and in vivo-favorable nanosize distribution were retained. This suggests that PFP can affect the size distribution of the droplets (Fig. 1f). Hyun et al. showed similar changes in size when PFP was encapsulated in echogenic glycol chitosan nanoparticles [32]. Additionally, the size of PCM-E2/PFPs showed no remarkable variation after 5 days of storage at 4 °C (Fig. 1g). The excellent stability of PCM-E2/PFPs during storage ensured their applicability during future experiments. The amount of E2 encapsulated in the PCM-E2/PFPs was determined using HPLC, with the encapsulation efficiency reaching 84.3 ± 2.8%.

**Fig. 2:**
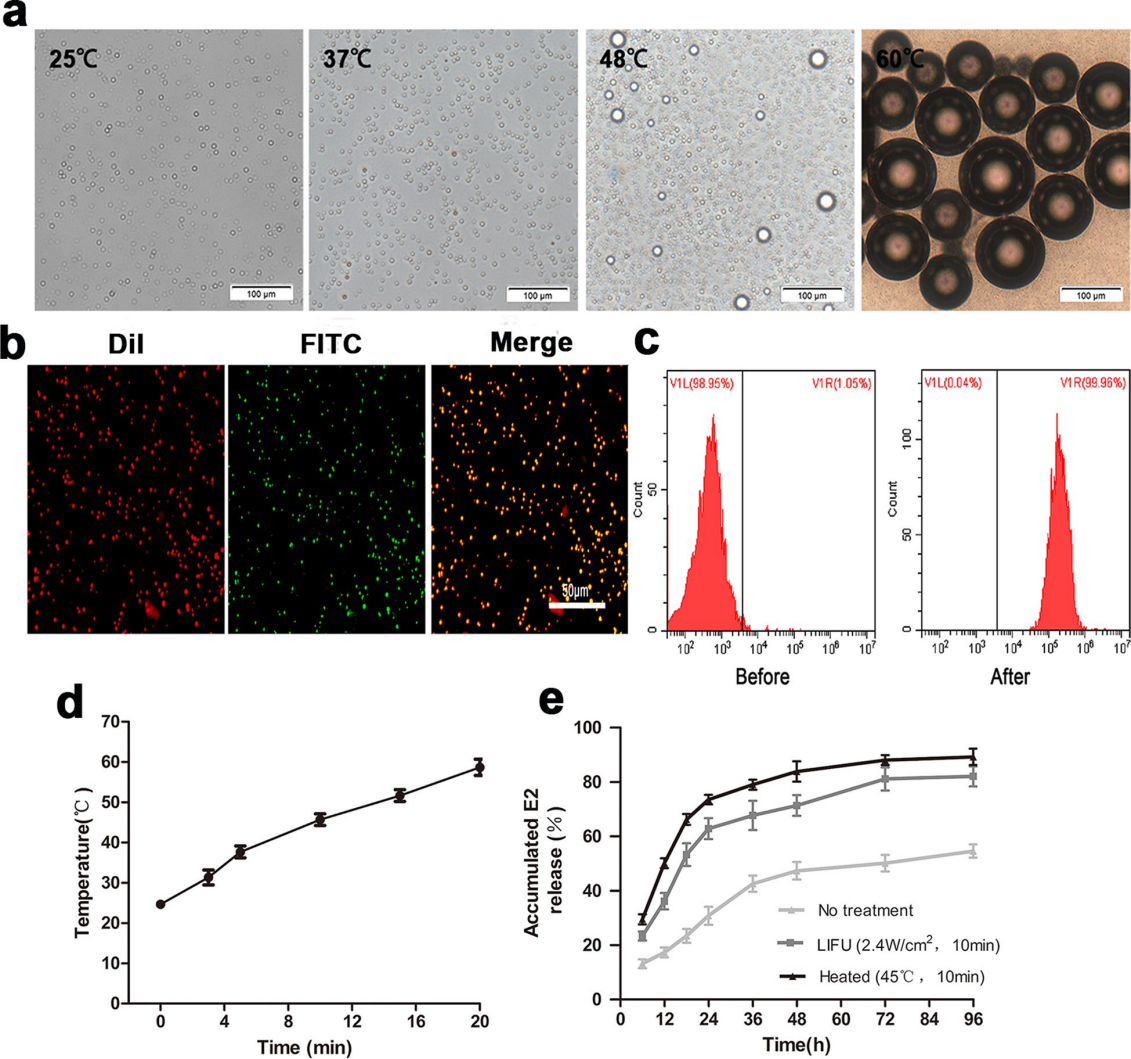
**a** Bubble formation behaviors of PCM-E2/PFPs on the external temperature changes from 25 to 60 °C. **b**, **c** CLSM image and flow-cytometry results showed preferential connection between FITC-labeled PCM peptide and DiI-labeled E2/PFPs nanodroplets. **d** The temperature change of PCM-E2/PFPs upon LIFU irradiation. **e** Cumulative release of E2 from PCM-E2/PFPs with/without LIFU or heated over time

Given that E2/PFPs nanoparticles do not have the ability to target cardiomyocytes on their own, they can accumulate at the cardiac site only through enhanced permeability. To improve their cardiomyocyte-targeting abilities, a 20-mer peptide with high binding affinity to cardiomyocytes was conjugated to the surface of the E2/PFPs. The extent of PCM conjugation was determined by detecting the connection between the FITC-labeled PCM and DiI-labeled nanodroplets. The merged orange images show a perfect connection between red E2/PFPs nanoprobes and green PCM peptides (Fig. 2b). The PCM conjugation efficiency was 97.33 ± 2.08% and accounted for only droplet-coupled PCM, given that all free conjugated nanodroplets were washed off (Fig. 2c).

### Temperature-dependent phase transition process of PCM-E2/PFPs

To evaluate the phase transition behavior of PCM-E2/PFPs, size variations at different temperatures were visualized using an inverted fluorescence microscope equipped with a temperature-controlled stage. The PCM-E2/PFPs expanded gradually with an increase in external temperature (Fig. 2a). At lower temperatures (25 and 37 °C), no noticeable microbubbles appeared in the images due to insufficient thermal energy needed to vaporize the nanodroplets, demonstrating that PCM-E2/PFPs had a robust structure. Nevertheless, the boiling point of PFP is 29 °C, theoretically rendering it liquid at room temperature but gaseous at body temperature. Interestingly, PCM-E2/PFPs still remained liquid at 37 °C without undergoing phase transition. This phenomenon can be attributed to the effects of Laplace pressure at the boundary of the nanodroplets, which retarded the gas release and allowed the nanodroplets to retain their initial state at 37 °C [33, 34]. Continuously increasing temperature to 48 °C resulted in an increase in PCM-E2/PFPs size, and the initiation of microbubble formation. Almost all particles gradually expanded, while a large number of bubbles were generated when the temperature was further elevated to 60 °C, indicating that external temperature is a critical factor in the phase transition process of PCM-E2/PFPs. It is worth mentioned that during the process of microbubble formation, adjacent bubbles tended to coalesce with each other and form larger ones, similar to that found in previous research [35]. The strong hydrophobic interaction among PFP gases in the core of the generated microbubbles can be a good explanation for this phenomenon, which promotes adherence among bubbles. Two appealing advantages can be suggested from the temperature-dependent behavior of PCM E2/PFPs. One is that the relative stability of the nanodroplets at 37 °C ensures prolonged circulation time in vivo, while another is that the robust polymer shell of the nanodroplets retards gas release, which is important for enhanced US imaging after LIFU irradiation.

### LIFU-triggered and temperature-dependent drug-release profile

To evaluate the temperature change of PCM-E2/PFPs triggered by the LIFU, the temperature were detected. As the trigger time of LIFU extended, the temperature gradually increased. When triggered for 10 min, the temperature reached to about 45 °C. When triggered for more than 10 min with LIFU, the temperature increase to 50 °C or even higher (Fig. 2d), which may result in the skin damage.

Given that PCM-E2/PFPs function as vessels for drug delivery, their drug-release profiles with and without LIFU exposure (2.4 W/cm^2^, 10 min) or heated (45 °C, 10 min) were verified. As expected, substantially higher E2 release rates were observed with LIFU-treated PCM-E2/PFPs (approximately 89% of E2) and with heated (approximately 82% of E2) than without any treatment (< 50% of E2) after 96 h (Fig. 2e). This indicated that external LIFU irradiation or heat treatment greatly enhanced the release of E2 from the nanodroplets. Meanwhile the E2 release rates was higher in LIFU-treated PCM-E2/PFPs than heat-treated PCM-E2/PFPs, this may due to the integral effect on all the nanodroplets, while LIFU, a focused ultrasound, may only function in small area. This is benefit for targeting drug release.

The high LIFU-triggered drug-release behaviors can maximize therapeutic efficacy through the expansion or rupture of the polymer shell. Considering the non-targeting nature of traditional non-focused ultrasonic devices and thermal damage from high-intensity focused ultrasound [36, 37], a LIFU-triggered drug delivery system could be an alternative method for promoting nanodroplet phase transition and drug release within the desired site. Similar to diagnostic US, LIFU can also generate acoustic waves outside the body and promote nanoprobe delivery to a specific organ.

### US imaging of PCM-E2/PFPs in vitro and in vivo

To better understand phase transition in PCM-E2/PFPs, the effect of frequency, a crucial factor for inducing phase transition in PFP-encapsulated nanodroplets, should be investigated comprehensively.

**Fig. 3:**
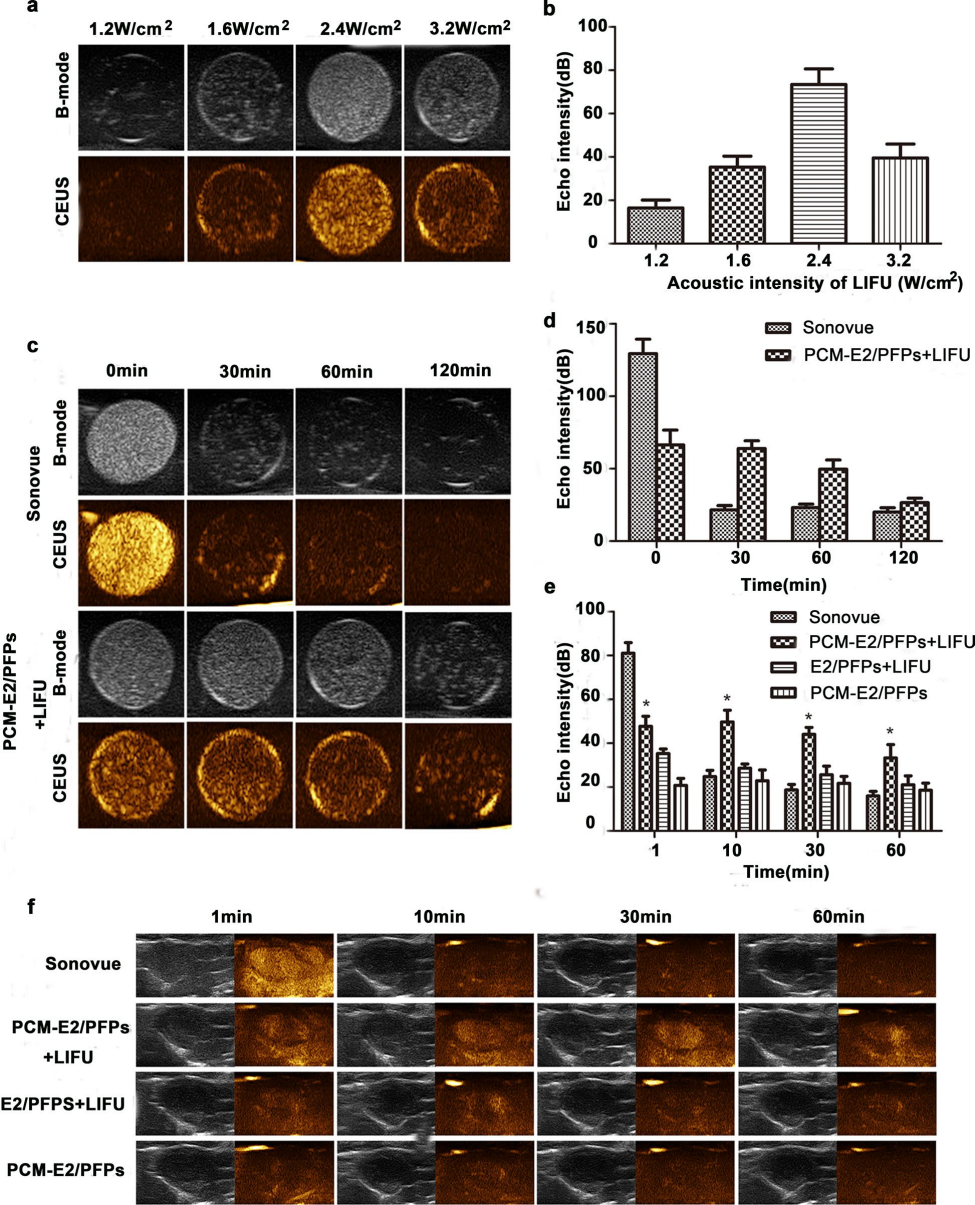
Echogenic properties of PCM-E2/PFPs in vitro and in vivo. **a**, **b** In vitro US images of PCM-E2/PFPs (1 mg/mL) in degassed water detected at various frequencies of LIFU (1.2, 1.6, 2.4 and 3.2 W/cm^2^). **c**, **d** In vitro US images of PCM-E2/PFPs at different imaging time points. **e**, **f** In vivo US imaging ability of PCM-E2/PFPs + LIFU compared to E2/PFPs + LIFU, PCM-E2/PFPs and Sonovue in the same experiment condition. *p < 0.05 vs PCM-E2/PFPs + LIFU group

Evaluation of the effect of LIFU frequency on US contrast imaging revealed that the images gradually brightened as frequency increased from 1.2 to 2.4 W/cm^2^. However, as LIFU frequency continuously elevated to 3.2 W/cm^2^, darkened images were observed. This probably indicated that the generated microbubbles had collapsed owing to the high frequency, which resulted in a remarkable decrease in the number of microbubbles (Fig. 3a). Furthermore, echo intensity analysis validated that the captured photographs were superior at a frequency of 2.4 W/cm^2^, which displayed the highest gray scale intensity (Fig. 3b). This result confirmed that LIFU frequency played an important role in improving phase transition by decreasing the droplet-to-bubble threshold. Therefore, 2.4 W/cm^2^ was the frequency selected for subsequent research, given that it was more suitable for PCM-E2/PFPs ultrasonography and prevented thermal injury to the skin. Moreover, after LIFU irradiation at 2.4 W/cm^2^, PCM-E2/PFPs were stable for more than 120 min in vitro (Fig. 3c), unlike the gas-filled sonovue solution, which was stable for only several minutes. These results demonstrated that PCM-E2/PFPs has great potential as an effective contrast agent for ultrasonic diagnosis.

Considering its outstanding performance during in vitro US imaging, the cardiac-targeting US imaging ability of PCM-E2/PFPs was confirmed in vivo by intravenous injection of targeting PCM-E2/PFPs and non-targeting E2/PFPs in rats. However, at an acoustic intensity of 2.4 W/cm^2^, no US contrast enhancement was found in the cardiac region (data not shown), which indicated insufficient energy for inducing phase transition of PCM-E2/PFPs within cardiac tissues. At a fixed LIFU acoustic intensity of 3.2 W/cm^2^, US imaging enhancement was apparent after irradiation for 10 min (data not shown). Therefore, in vivo US imaging experiments were performed at 3.2 W/cm^2^ for 10 min. We investigated the post-injection imaging performance of groups with and without LIFU stimulus. The results showed no obvious differences in US imaging between the PCM-E2/PFPs and E2/PFPs + LIFU groups. Nevertheless, LIFU-triggered PCM-E2/PFPs showed markedly enhanced capability for US imaging. Moreover, the change in echo intensity from 48.01 ± 7.94 to 33.68 ± 10.3 within 60 min during cardiac US imaging (Fig. 3e, f) indicated that LIFU can enhance the US imaging capability of PCM-E2/PFPs and thereby improve its accuracy during cardiac diagnosis. We also found that quantitative echo intensity values were substantially higher in the PCM-E2/PFPs + LIFU group than in the E2/PFPs + LIFU group (Fig. 3e), indicating effective cardiac accumulation. Primers also showed that acoustic nanodroplets were able to detect abnormalities in myocardial perfusion. Nevertheless, further studies are needed to optimize these nanodroplets in order to lower their vaporization threshold in vivo. This would increase nanodroplets vaporization in targeted tissues given the relatively lower imaging enhancement observed in nanodroplets than in microbubbles despite injecting greater amounts thereof.

### In vivo biodistribution of PCM-E2/PFPs in rats

**Fig. 4:**
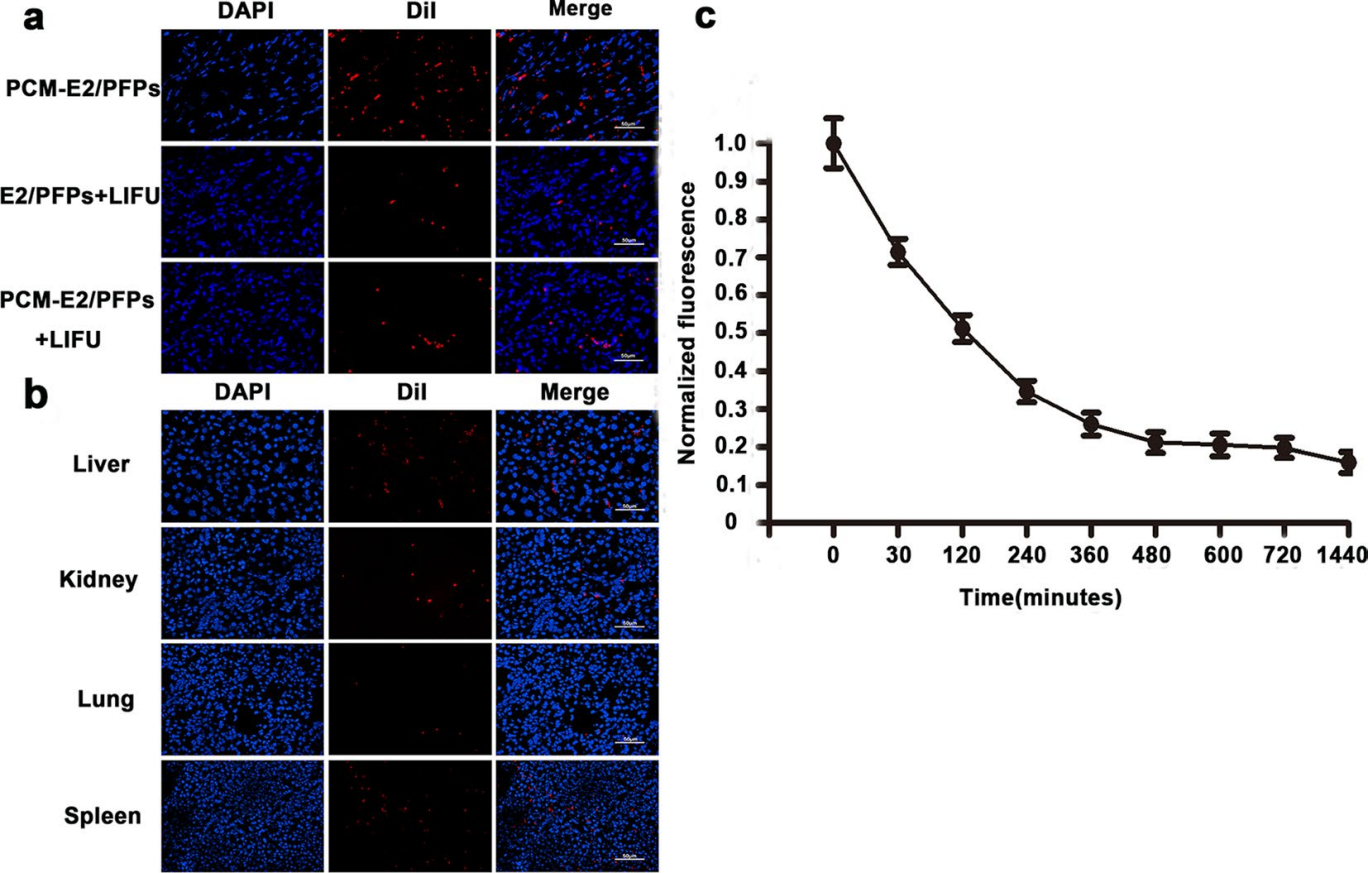
Biopanning of PCM-E2/PFPs delivery in vivo. **a**, **b** CLSM analysis of cardiac sections for the experimental groups and substantial localization in other tissues such as kidney, liver, spleen and lungs from rats injected PCM-E2/PFPs nanodroplets with LIFU exposure. **c** The blood circulation of PCM-E2/PFPs after LIFU exposure groups at different post-injection time

The targeted transportation and distribution of PCM-E2/PFPs in vivo were determined using DiI-labeled nanodroplets. Prominent and wide-ranging red dots representing DiI-labeled PCM-E2/PFPs distribution were observed in the cardiac cryosections in the PCM-E2/PFPs + LIFU group than PCM-E2/PFPs and E2/PFPs + LIFU groups under CLSM 12 h after injection, suggesting excellent cardiac targeting. Moreover, cardiac nanodroplet accumulation in the PCM-E2/PFPs + LIFU group was more prominent (Fig. 4a) than other tissues (liver, kidney, lung, spleen) (Fig. 4b), given that LIFU can be focused and can penetrate nanodroplets deep within the target regions. In addition, fluorometric analysis of DiI signals showed a 50% reduction in DiI serum concentration within 30 min of injection in the PCM-E2/PFPs + LIFU group and that the DiI signal lasted for more than 24 h (Fig. 4c). These results suggest that the combination of PCM-E2/PFPs with LIFU could greatly improve the efficiency of drug delivery in terms of PCM-guided active targeting, LIFU-triggered passive targeted drug release, and cavitation-induced enhancement of vessel permeability.

### Assessment of safety

Histopathological evaluation of major organs, including the lungs, liver, spleen, kidneys, and brain, after PCM-E2/PFPs + LIFU treatment was performed using HE staining. As shown in Additional file 1: Fig. S1a, no noticeable morphological abnormalities in tissue architecture were detected in the PCM-E2/PFPs + LIFU group. To further assess the biosafety of this synergistic strategy, blood biochemical tests, including those for liver and renal function, were carried out. No significant variance in biochemical indicators of liver and kidney function were found among any group (Additional file 1: Fig. S1b), indicating excellent biocompatibility of PCM-E2/PFPs in rats. This suggested that PCM-E2/PFPs may have the potential to effectively reduce the side effects of E2.

### Prevention of LV dysfunction in rats with cardiac hypertrophy

**Fig. 5:**
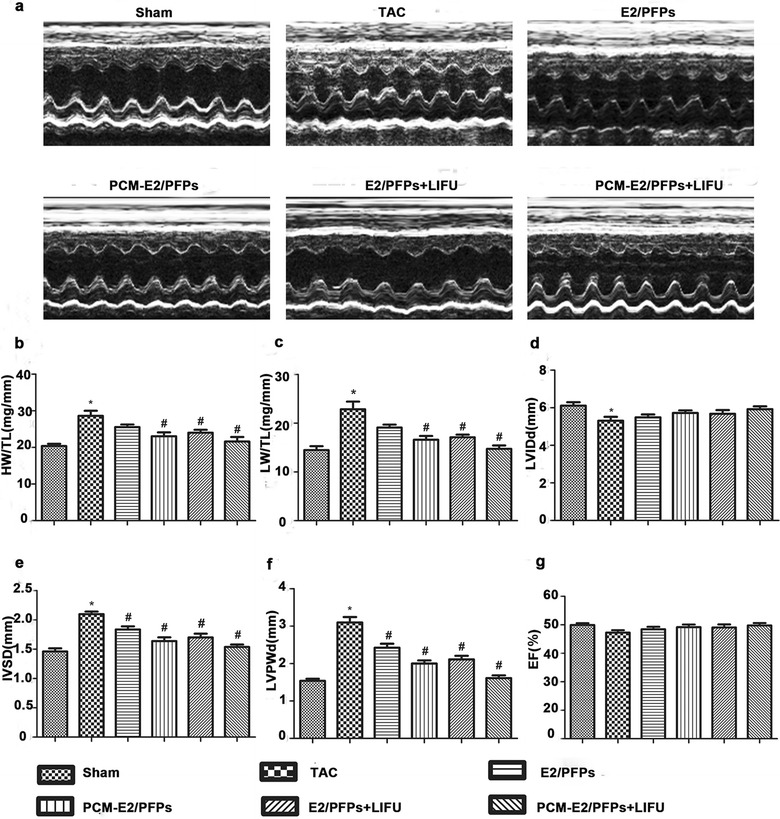
M-mode echocardiographic analysis in experiment rats. **a** Representative echocardiographic images (n = 8, each group). **b**, **c** The ratio of heart weight (HW) and left ventricle weight (LVW) to tibia length (TL). **d**–**g** The changes in LV diastolic internal dimension (LVDD), interventricular septum end diastolic thickness (IVSd), posterior wall thickness at the end-diastole (LVPWd), and LV ejection fraction (EF), determined by echocardiography. *p < 0.05 vs sham group; ^#^p < 0.05 vs TAC group; ^+^p < 0.05 vs PCM-E2/PFPs + LIFU group

During the study, no morality of the animals was observed. Compared to the sham group, the TAC rats exhibited a significant increase in HM/TL, LM/TL, LVPWd, and IVSD, but a decrease in LVDD, indicating the occurrence of cardiac hypertrophy (Fig. 5a–g). In general, all four E2-treated groups exhibited slightly better LVDD and significantly higher HM/TL, LW/TL, LVPWd, and IVSD compared to the untreated hypertrophic animals (p < 0.05). The results indicated that E2 treatment attenuates cardiomyopathy. Furthermore, the greatest differences in the five parameters above were observed in PCM-E2/PFPs + LIFU group. In comparison, LM/TL and LVPWd were much lower in the PCM-E2/PFPs + LIFU group than in other groups (p < 0.05). LVEF, as determined by echocardiography, was similar in all groups, indicating that LV function remained compensated in all groups with TAC surgery.

**Fig. 6:**
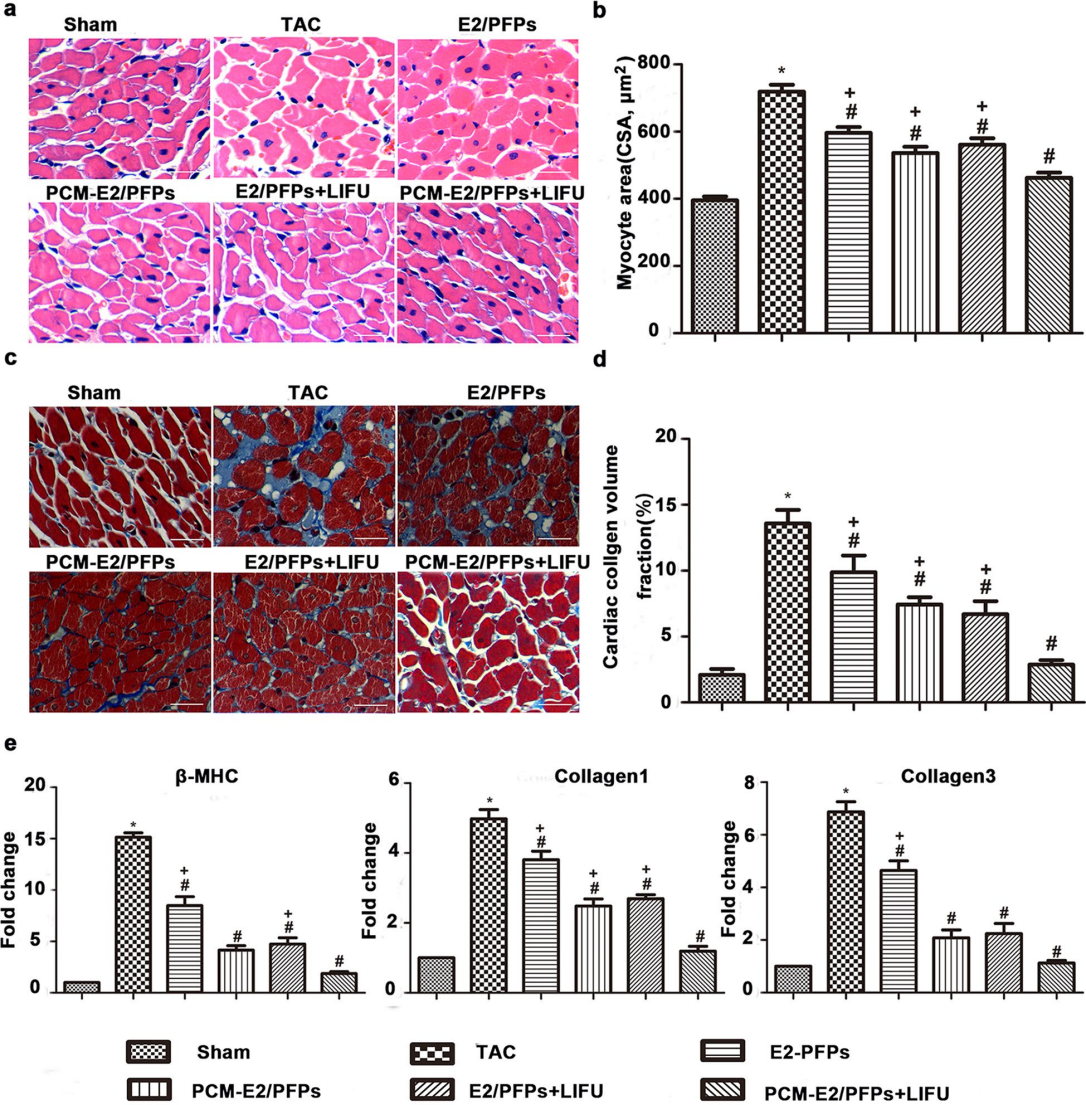
Regression of cardiac hypertrophy in experiment rats. **a**, **c** Representative pictures of cardiac tissue cross-sections with haematoxylin/eosin (H/E) staining and Masson trichrome staining from different treatment groups (n = 8, each group, scale bar = 50 μm, magnification = ×400). **b**, **d** Quantitative analysis of average cross sectional area of myocytes (CSA) in cardiac tissues and cardiac collagen volume fraction (CVF). **e** Quantitative RT-PCR analysis of the hypertrophy-related genes β-MHC and fibrosis markers Collagen 1 and Collagen 3. GAPDH was used as an internal loading control. *p < 0.05 vs sham group; ^#^p < 0.05 vs TAC group; ^+^p < 0.05 vs PCM-E2/PFPs + LIFU group

Histological analyses of HE and Masson’s staining were performed using paraffin-embedded cardiac tissues (Fig. 6a, c). Cardiomyocyte disorganization and hypertrophy were accompanied by an altered collagen network structure in the studied animals. Moreover, CSA and CVF were significantly higher in the TAC group than in sham and other treated groups. Compared to the TAC group (719.08 ± 93.19 μm^2^ and 13.58 ± 2.05%), CSA and CVF were significantly lower in the E2/PFPs (596.45 ± 79.87 μm^2^ and 9.9 ± 2.48%), E2/PFPs + LIUF (561.17 ± 88.57 μm^2^ and 7.45 ± 1.08%), and PCM-E2/PFPs (536.27 ± 85.07 μm^2^ and 6.7 ± 1.98%) groups. Furthermore, CSA and CVF were significantly lower in the PCM-E2/PFPs + LIFU group (462.31 ± 74.04 μm^2^ and 2.88 ± 0.67%) than in other treatment groups (Fig. 6b, d).

RT-PCR analysis clearly showed that cardiac tissue-targeted delivery of therapeutic payloads combined with LIFU irradiation significantly regressed the cardiac hypertrophy as evidenced by the reduced expression of hypertrophy markers (Fig. 6e) and the higher expression of β-MHC, Collagen 1, and Collagen 3 in TAC group compared to the sham group (p < 0.05). Remarkably, E2-treated groups showed significantly lower expression levels of β-MHC, Collagen 1, and Collagen 3 than the TAC group. Moreover, the PCM-E2/PFPs + LIFU group exhibited the lowest expression of β-MHC, Collagen 1, and Collagen 3 among the treatment groups (p < 0.05).

Therapeutic efficacy data showed that LIFU-irradiated PCM-E2/PFPs might have increased the local concentration of the released drug in cardiac tissues, maximizing its anti-hypertrophic efficacy. Theranostic approaches have attracted major attention, given that they allow simultaneous diagnosis and treatment. The current study is the first to report on the development of a theranostic E2-loaded droplet-to-bubble nanoprobe for cardiac-targeted imaging and treatment. Three main factors may explain the synergistic mechanism through which PCM-E2/PFPs + LIFU irradiation affects cardiac hypertrophy. First, the excellent targeting ability of PCM peptides and cavitation-induced enhancement of vessel permeability increased the accumulation of nanodroplets in cardiac tissues. Second, LIFU-triggered passive targeted drug release promotes E2 release from PCM-E2/PFPs, accelerating E2 accumulation in cardiac tissues while minimizing systemic toxicity. The third factor may be attributed to the effects of myocardial cavitation-enabled therapy (MCET). Myocardial contrast echocardiography has been shown to be capable of causing lethal injury to cardiomyocytes, resulting in scattered microlesions throughout the scanned region. Interestingly, these microlesions can innocuously heal within a few weeks with minimal scarring, leaving a marked reduction in tissue volume [38], This noninvasive and relatively gentle method of tissue reduction has been shown to be advantageous in the treatment of cardiac hypertrophy [39]. Therefore, we hypothesis that this “droplets-to-bubbles” nanodroplets will have the similar effects on hypertrophic heart, which may be one potencial mechanism of PCM-E2/PFPs with LIFU irradiation in preventing myocardial hypertrophy, the precise MCET of PCM-E2/PFPs need to further be testified.

Taken together, our study has provided extensive evidence to strongly suggest that PCM-E2/PFPs combining with LIFU technique have a great potential in facilitating targeted imaging and delivery of E2 for the prevention of cardiac hypertrophy thus minimizing adverse effects to other organs.

## Conclusion

In summary, we successfully prepared PCM-conjugated and E2-loaded acoustic nanodroplets and demonstrated their potential use in targeted diagnosis and therapy on pathological myocardium. Conventional treatment with E2 against cardiac ailments has been reported to improve cardiac function considerably, yet leads to severe adverse all over the body. Such a newly developed nanoconstruct thus promises to be a potential clinical tool for off-target therapeutics delivery as well as ultrasound contrast enhancers for theranostics on myocardial pathophysiology.

## Additional files

**Additional file 1: Fig. S1.** Safety evaluation of PCM-E2/PFPs with LIFU. (a) H&E staining of various organs of hypertrophic rats after treatment with LIFU in each group; (b) Blood biochemical examination ofliver function, and renal function after treatment. N=3.

**Additional file 2.** Additional tables.

## Abbreviations

E2: 17β-estradiol
LV: left ventricular
US: ultrasound
PFP: perfluoropentane
PCM: primary cardiomyocyte specific peptide
LIFU: low-intensity focused ultrasound
OVX: ovariectomy
TAC: transverse aortic constriction
CSA: cross sectional area
CVF: collagen volume fraction
HW: hearts weights
LW: left ventricle weights
IVSD: interventricular septum end-diastolic thickness
LVPWd: posterior end-diastolic wall thickness
LVDD: end-diastolic diameter
EF: ejection fraction
ALT: alanine transaminase
ALP: alkaline phosphates
AST: aspartate aminotransferase
Cr: creatinine
BUN: blood urea nitrogen
UA: uric acid
