# Supplementary figures and images for: Cardiomyocyte-targeted and 17β-estradiol-loaded acoustic nanoprobes as a theranostic platform for cardiac hypertrophy
Source: J Nanobiotechnology. 2018 Mar 30;16:36. doi: 10.1186/s12951-018-0360-3 (PMC5877324; doi:10.1186/s12951-018-0360-3)

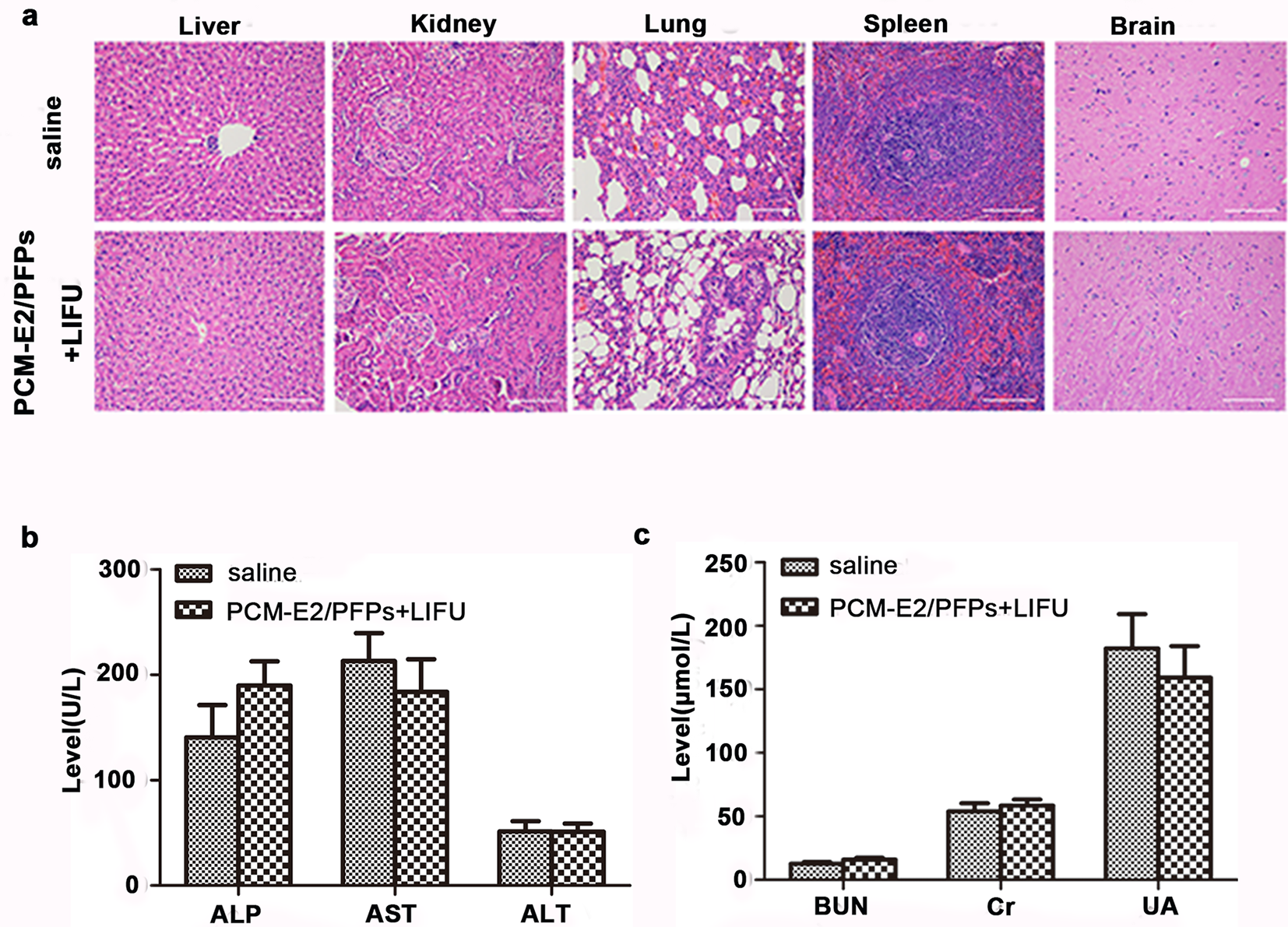

Supplement: Supplementary file 1 — Additional file 1: Fig. S1. Safety evaluation of PCM-E2/PFPs with LIFU. (a) H&E staining of various organs of hypertrophic rats after treatment with LIFU in each group; (b) Blood biochemical examination ofliver function, and renal function after treatment. N=3. [file 12951_2018_360_MOESM1_ESM.tif]
