# Supplementary material for: Cardiomyocyte-targeted and 17β-estradiol-loaded acoustic nanoprobes as a theranostic platform for cardiac hypertrophy
Source: J Nanobiotechnology. 2018 Mar 30;16:36. doi: 10.1186/s12951-018-0360-3 (PMC5877324; doi:10.1186/s12951-018-0360-3)
Supplement: Supplementary file 2 — Additional file 2. Additional tables. [file 12951_2018_360_MOESM2_ESM.docx]

Fig. 1d, 1e

| Samples | size(nm) | Zeta potencial(-mv) |
| --- | --- | --- |
| 1 | 417 | -21 |
| 2 | 408 | -19 |
| 3 | 430 | -18 |

Fig. 1g

| Time(days) | Sanples 1(nm) | Sanples 2(nm) | Sanples 3(nm) |
| --- | --- | --- | --- |
| 0.5 | 412 | 408 | 430 |
| 1 | 412 | 408 | 435 |
| 2 | 420 | 418 | 437 |
| 3 | 431 | 444 | 454 |
| 4 | 468 | 480 | 527 |
| 5 | 555 | 563 | 591 |

Fig. 2d

| Time(min) | temperature(°C) | | |
| --- | --- | --- | --- |
| 0 | 25 | 24 | 25 |
| 3 | 29 | 35 | 30 |
| 5 | 35 | 40 | 38 |
| 10 | 43 | 46 | 48 |
| 15 | 49 | 52 | 54 |
| 20 | 55 | 62 | 59 |

Fig. 2e

| Times  (hours) | NO treatment(％) | | | LIFU(％) | | | Heat(％) | | |
| --- | --- | --- | --- | --- | --- | --- | --- | --- | --- |
| 6 | 12.21 | 10.93 | 16.3 | 23.27 | 20.47 | 26.27 | 33.14 | 28.11 | 27.23 |
| 12 | 14.63 | 16.49 | 20.79 | 30.41 | 37.58 | 40.47 | 53.25 | 47.01 | 50.18 |
| 18 | 18.77 | 24.06 | 27.48 | 50.95 | 47.51 | 61.42 | 63.85 | 70.17 | 64.67 |
| 24 | 28.69 | 37.31 | 26.45 | 56.21 | 62.77 | 69.57 | 73.68 | 76.42 | 70.43 |
| 36 | 38.72 | 40.66 | 48.47 | 60.52 | 64.43 | 78.19 | 82.39 | 76.01 | 78.65 |
| 48 | 44.75 | 43.49 | 53.79 | 64.74 | 71.47 | 77.92 | 76.84 | 89.67 | 85.1 |
| 72 | 49.13 | 55.89 | 45.47 | 79.36 | 89.17 | 74.84 | 87.23 | 91.48 | 85.42 |
| 96 | 52.95 | 59.44 | 51.43 | 80.01 | 89.23 | 76.97 | 83.26 | 91.54 | 92.93 |

Fig. 3b

| Frequency | 0.8W/cm^2^(dB) | 1.2W/cm^2^(dB) | 2.4 W/cm^2^(dB) | 3.2 W/cm^2^(dB) |
| --- | --- | --- | --- | --- |
| Sample1 | 23.2 | 25.11 | 85.42 | 50.76 |
| Sample2 | 10.6 | 41.3 | 60.75 | 28.49 |
| Sample3 | 15.56 | 39.4 | 74.28 | 39.25 |

Fig. 3d

| samples | Sonovue(dB) | | | PCM-E2/PFPs+LIFU(dB) | | |
| --- | --- | --- | --- | --- | --- | --- |
| 0min | 138.29 | 109.54 | 140.6 | 50.14 | 64.15 | 85.19 |
| 30min | 27.29 | 20.15 | 17.26 | 72.59 | 65.15 | 54.19 |
| 60min | 23.65 | 18.62 | 27.16 | 49.76 | 60.68 | 38.59 |
| 120min | 21.84 | 14.29 | 24.15 | 29.11 | 30.15 | 20.57 |

Fig. 3e

| Times  (min) | Sonovue | | | PCM-E2/PFPs+LIFU | | | E2/PFPs+LIFU | | | PCM-E2/PFPs | | |
| --- | --- | --- | --- | --- | --- | --- | --- | --- | --- | --- | --- | --- |
| 1 | 73.69 | 90.12 | 79.25 | 40.56 | 47.12 | 56.36 | 32.54 | 35.18 | 39.46 | 26.38 | 20.64 | 15.45 |
| 10 | 23.65 | 20.58 | 30.16 | 42.15 | 60.16 | 47.18 | 31.23 | 30.26 | 25.18 | 26.38 | 29.12 | 13.23 |
| 30 | 23.65 | 15.26 | 17.18 | 39.26 | 50.15 | 43.34 | 30.16 | 18.79 | 29.58 | 21.84 | 27.15 | 16.21 |
| 60 | 20.02 | 13.26 | 14.59 | 25.56 | 30.21 | 45.26 | 29.26 | 15.2 | 19.1 | 23.65 | 12.45 | 19.48 |

Fig. 4c

| Time(min) | Sample1(dB) | Sample2 (dB) | Sample3  (dB) | Sample4 (dB) | Sample5 (dB) |
| --- | --- | --- | --- | --- | --- |
| 0 | 1.14 | 0.95 | 0.85 | 0.89 | 1.17 |
| 30 | 0.76 | 0.71 | 0.61 | 0.68 | 0.81 |
| 120 | 0.55 | 0.54 | 0.38 | 0.5 | 0.59 |
| 240 | 0.33 | 0.32 | 0.28 | 0.45 | 0.35 |
| 360 | 0.35 | 0.26 | 0.3 | 0.19 | 0.2 |
| 480 | 0.28 | 0.26 | 0.18 | 0.13 | 0.21 |
| 600 | 0.24 | 0.26 | 0.13 | 0.26 | 0.14 |
| 720 | 0.27 | 0.24 | 0.13 | 0.2 | 0.15 |
| 1440 | 0.26 | 0.18 | 0.13 | 0.12 | 0.11 |

Fig. 5b (HW/TL)

| samples | Sham  (mg/mm) | TAC  (mg/mm) | E2/PFPs  (mg/mm) | PCM-E2/PFPs  (mg/mm) | E2/PFPs+LIFU  (mg/mm) | PCME2/PFPs+LIFU  (mg/mm) |
| --- | --- | --- | --- | --- | --- | --- |
| 1 | 18.38 | 27.75 | 28.72 | 25.28 | 26.38 | 23.25 |
| 2 | 20.85 | 33.84 | 27.64 | 24.77 | 24.17 | 21.65 |
| 3 | 19.65 | 28.74 | 26.43 | 23.58 | 27.31 | 25.24 |
| 4 | 21.62 | 25.70 | 24.62 | 19.18 | 22.53 | 18.46 |
| 5 | 21.36 | 27.01 | 25.49 | 22.42 | 21.86 | 19.52 |

Fig. 5c (LW/TL)

| samples | Sham  (mg/mm) | TAC  (mg/mm) | E2/PFPs  (mg/mm) | PCM-E2/PFPs  (mg/mm) | E2/PFPs+LIFU  (mg/mm) | PCM-E2/PFPs+LIFU  (mg/mm) |
| --- | --- | --- | --- | --- | --- | --- |
| 1 | 12.11 | 22.50 | 17.82 | 19.03 | 18.43 | 15.05 |
| 2 | 15.01 | 28.89 | 21.16 | 17.70 | 17.41 | 13.62 |
| 3 | 13.18 | 19.53 | 22.61 | 15.46 | 19.47 | 16.69 |
| 4 | 16.44 | 21.64 | 18.60 | 14.90 | 16.79 | 13.21 |
| 5 | 15.72 | 21.76 | 19.35 | 15.97 | 17.12 | 14.57 |

Fig. 5d (LVIDd)

| samples | Sham  (mm) | TAC  (mm) | E2/PFPs  (mm) | PCM-E2/PFPs  (mm) | E2/PFPs+LIFU  (mm) | PCM-E2/PFPs+LIFU  (mm) |
| --- | --- | --- | --- | --- | --- | --- |
| 1 | 6.50 | 5.20 | 5.40 | 5.70 | 5.90 | 5.70 |
| 2 | 5.90 | 5.40 | 5.20 | 5.40 | 6.10 | 6.00 |
| 3 | 6.50 | 6.50 | 5.00 | 6.10 | 5.00 | 5.70 |
| 4 | 5.50 | 4.80 | 5.40 | 5.20 | 5.70 | 6.90 |
| 5 | 5.20 | 4.80 | 6.00 | 5.90 | 6.60 | 5.90 |
| 6 | 6.50 | 5.50 | 5.20 | 5.50 | 5.90 | 5.50 |
| 7 | 6.50 | 4.80 | 6.30 | 6.30 | 5.20 | 6.00 |
| 8 | 6.30 | 5.50 | 5.40 | 5.70 | 5.00 | 5.70 |

Fig. 5e (LVSD)

| samples | Sham  (mm) | TAC  (mm) | E2/PFPs  (mm) | PCM-E2/PFPs  (mm) | E2/PFPs+LIFU  (mm) | PCM-E2/PFPs+LIFU  (mm) |
| --- | --- | --- | --- | --- | --- | --- |
| 1 | 1.40 | 1.90 | 2.10 | 1.90 | 2.00 | 1.50 |
| 2 | 1.50 | 2.20 | 1.60 | 2.00 | 1.60 | 1.70 |
| 3 | 1.60 | 2.10 | 1.90 | 1.80 | 1.70 | 1.40 |
| 4 | 1.20 | 2.30 | 2.10 | 1.90 | 1.90 | 1.40 |
| 5 | 1.70 | 2.10 | 1.60 | 1.60 | 1.80 | 1.50 |
| 6 | 1.50 | 2.10 | 1.90 | 1.90 | 1.50 | 1.50 |
| 7 | 1.40 | 2.10 | 1.70 | 1.60 | 1.80 | 1.60 |
| 8 | 1.40 | 2.00 | 1.80 | 1.70 | 2.10 | 1.70 |

Fig. 5f (LVPWd)

| samples | Sham  (mm) | TAC  (mm) | E2/PFPs  (mm) | PCM-E2/PFPs  (mm) | E2/PFPs+LIFU  (mm) | PCM-E2/PFPs+LIFU  (mm) |
| --- | --- | --- | --- | --- | --- | --- |
| 1 | 1.50 | 2.80 | 2.40 | 2.20 | 2.40 | 1.60 |
| 2 | 1.30 | 3.50 | 2.30 | 1.80 | 2.00 | 1.80 |
| 3 | 1.50 | 3.40 | 2.20 | 2.30 | 2.50 | 1.40 |
| 4 | 1.50 | 3.10 | 2.80 | 2.20 | 2.30 | 1.60 |
| 5 | 1.40 | 2.50 | 2.10 | 1.80 | 1.90 | 1.40 |
| 6 | 1.60 | 3.40 | 2.10 | 1.80 | 1.80 | 1.40 |
| 7 | 1.70 | 3.50 | 2.70 | 2.20 | 2.20 | 1.90 |
| 8 | 1.80 | 2.60 | 2.80 | 1.70 | 1.80 | 1.80 |

Fig. 5g (EF)

| samples | Sham  (％) | TAC  (％) | E2/PFPs  (％) | PCM-E2/PFPs(％) | E2/PFPs+LIFU  (％) | PCM-E2/PFPs+LIFU  (％) |
| --- | --- | --- | --- | --- | --- | --- |
| 1 | 74.0 | 76.0 | 72.0 | 82.0 | 86.0 | 82.0 |
| 2 | 77.0 | 74.0 | 76.0 | 70.0 | 82.0 | 78.0 |
| 3 | 79.0 | 82.0 | 78.0 | 76.0 | 73.0 | 79.0 |
| 4 | 80.0 | 70.0 | 70.0 | 76.0 | 82.0 | 71.0 |
| 5 | 80.0 | 70.0 | 78.0 | 74.0 | 67.0 | 86.0 |
| 6 | 81.0 | 81.0 | 83.0 | 83.0 | 81.0 | 83.0 |
| 7 | 82.0 | 70.0 | 84.0 | 84.0 | 76.0 | 82.0 |
| 8 | 86.0 | 73.0 | 74.0 | 82.0 | 78.0 | 75.0 |

Fig. 6b (myocyte area(CSA))

| samples | Sham  (μm^2^) | TAC  (μm^2^) | E2/PFPs  (μm^2^) | PCM-E2/PFPs  (μm^2^) | E2/PFPs+LIFU  (μm^2^) | PCM-E2/PFPs+LIFU  (μm^2^) |
| --- | --- | --- | --- | --- | --- | --- |
| 1 | 403.167 | 704.000 | 558.389 | 550.167 | 600.278 | 353.278 |
| 2 | 314.444 | 670.889 | 496.778 | 532.222 | 684.222 | 496.111 |
| 3 | 314.000 | 731.667 | 760.389 | 587.889 | 550.167 | 488.333 |
| 4 | 440.111 | 777.944 | 532.222 | 414.167 | 524.056 | 366.833 |
| 5 | 376.500 | 681.556 | 587.889 | 594.944 | 559.278 | 432.278 |
| 6 | 393.8890 | 895.722 | 616.333 | 436.556 | 527.389 | 498.556 |
| 7 | 317.500 | 774.611 | 594.944 | 640.111 | 441.722 | 362.778 |
| 8 | 395.111 | 692.222 | 655.889 | 497.944 | 688.889 | 412.389 |
| 9 | 328.889 | 616.333 | 622.222 | 575.278 | 552.889 | 366.556 |
| 10 | 353.278 | 882.833 | 497.944 | 587.167 | 574.889 | 544.833 |
| 11 | 436.556 | 658.222 | 575.278 | 515.056 | 379.167 | 488.611 |
| 12 | 395.2780 | 819.333 | 587.167 | 549.556 | 631.222 | 441.111 |
| 13 | 376.778 | 759.333 | 615.944 | 488.222 | 428.222 | 463.722 |
| 14 | 373.278 | 671.278 | 733.944 | 593.611 | 587.278 | 328.8890 |
| 15 | 422.083 | 737.139 | 549.556 | 427.889 | 576.778 | 514.222 |
| 16 | 425.2220 | 665.722 | 594.833 | 588.444 | 537.056 | 569.167 |
| 17 | 494.722 | 550.667 | 593.611 | 596.722 | 712.500 | 573.278 |
| 18 | 491.722 | 725.056 | 427.889 | 739.000 | 657.500 | 551.833 |
| 19 | 426.111 | 712.667 | 588.444 | 482.611 | 525.556 | 480.333 |
| 20 | 388.2817 | 541.222 | 596.722 | 491.972 | 603.833 | 512.278 |

Fig. 6d cardiac collagen volume fraction (％)

| samples | Sham  **(％)** | TAC  **(％)** | E2/PFPs  **(％)** | PCM-E2/PFPs**(％)** | E2/PFPs+LIFU  **(％)** | PCM-E2/PFPs  +LIFU**(％)** |
| --- | --- | --- | --- | --- | --- | --- |
| 1 | 1.5 | 11.8 | 7.5 | 4.4 | 6.0 | 3.6 |
| 2 | 2.4 | 12.1 | 10.3 | 7.8 | 8.6 | 2.8 |
| 3 | 3.2 | 16.2 | 13.2 | 5.8 | 7.6 | 3.1 |
| 4 | 1.3 | 14.2 | 8.6 | 8.8 | 7.6 | 2. |

Fig. 6e (β-MHC)

| Samples | sham | TAC | E2/PFPs | PCM-E2/PFPs | E2/PFPs  +LIFU | PCM-E2/PFPs  +LIFU |
| --- | --- | --- | --- | --- | --- | --- |
| 1 | 1. | 14.487380 | 10.176700 | 4.771154 | 4.834067 | 1.541726 |
| 2 | 1. | 15.003140 | 7.326388 | 4.396124 | 3.666010 | 1.901803 |
| 3 | 1. | 15.938510 | 7.984732 | 3.332234 | 5.748149 | 2.165928 |

Collagen 1

| Samples | sham | TAC | E2/PFPs | PCM-E2/PFPs | E2/PFPs+LIFU | PCM-E2/PFPs+LIFU |
| --- | --- | --- | --- | --- | --- | --- |
| 1 | 1. | 5.018131 | 3.791252 | 2.619100 | 2.474611 | 0.9414815 |
| 2 | 1. | 5.417835 | 3.397734 | 2.084373 | 2.862960 | 1.219041 |
| 3 | 1. | 4.500012 | 4.236035 | 2.748964 | 2.740263 | 1.416497 |

Collagen 3

| Samples | sham | TAC | E2/PFPs | PCM-E2/PFPs | E2/PFPs+LIFU | PCM-E2/PFPs+LIFU |
| --- | --- | --- | --- | --- | --- | --- |
| 1 | 1. | 6.449193 | 4.117969 | 1.849078 | 2.200663 | 1.316121 |
| 2 | 1. | 6.511074 | 5.357886 | 1.715970 | 1.596835 | 1.065991 |
| 3 | 1. | 7.647927 | 4.445647 | 2.682145 | 2.932346 | 0.9963021 |

Fig. sb

|  | Saline | | | | | PCM-E2/PFPs+LIFU | | | | |
| --- | --- | --- | --- | --- | --- | --- | --- | --- | --- | --- |
| ALP | 256.40 | 151.40 | 105.20 | 96.20 | 93.837 | 264.10 | 210.2000 | 184.0 | 164.70 | 126.50 |
| AST | 155.90 | 283.80 | 213.90 | 153.00 | 259.500 | 205.10 | 107.9000 | 290.0 | 173.30 | 143.10 |
| ALT | 43.58 | 86.35 | 58.29 | 34.57 | 34.040 | 46.35 | 35.7500 | 79.3 | 53.89 | 40.57 |

Fig. sc

|  | Saline | | | | | PCM-E2/PFPs+LIFU | | | | |
| --- | --- | --- | --- | --- | --- | --- | --- | --- | --- | --- |
| BUN | 16.25 | 15.4 | 10.68 | 9.25 | 11.75 | 20.88 | 16.89 | 16.07 | 12.02 | 14.22 |
| Cr | 51.30 | 78.5 | 49.30 | 48.50 | 42.00 | 53.30 | 68.90 | 70.45 | 45.89 | 54.00 |
| UA | 129.90 | 124.4 | 196.60 | 273.40 | 186.50 | 187.40 | 97.40 | 115.40 | 235.40 | 160.60 |
